# Self‐selecting peer groups formed within the laboratory environment have a lasting effect on individual student attainment and working practices

**DOI:** 10.1002/2211-5463.12902

**Published:** 2020-06-07

**Authors:** Melissa M. Lacey, Susan G. Campbell, Holly Shaw, David P. Smith

**Affiliations:** ^1^ Department of Biosciences and Chemistry Sheffield Hallam University UK

**Keywords:** attainment, laboratory, learning space, peer effects, peer groups, performance

## Abstract

Within the present study, we investigate the lasting effect of laboratory peer group interactions on the end of year attainment of Biosciences and Chemistry students. By asking students to identify who they primarily work with within the laboratory environment and evaluating the interactions through cluster analysis, we identified two main categories of laboratory peer groups: the first long‐lived well‐established pairings of two students, ‘swans’, who work together for all or the majority of the laboratory sessions; and the second dynamic fluid groups, ‘dolphins’, of between three and nine students who work with each other interchangeably. Statistical analysis is presented, which demonstrates that individuals within each laboratory peer group were likely to achieve a similar average mark at the end of the first year of study on the course. We identified the driving factors for the formation of these groups as friendship and perceived work ethic. There is a preference for high‐achieving students to work with other high‐achieving students and lower‐achieving to group around a shared social background. Targeted interventions, in which pairings were selected by the tutor at the onset of the study, altered the ratio from long‐lived pairs to more dynamic groups and increased students’ willingness to work with others outside of their group but did not change the drivers of group formation or resulting pattern of achievement. We conclude with recommendations around group working within the laboratory environment.

## Graphical abstract

Here, we investigate the effect of laboratory peer group interactions on end of year attainment. We identified two major group types: ‘swans’, pairs of students who work together all the time; and ‘dolphins’, extended networks who work with each other interchangeably. Individuals are shown to gain similar marks to their laboratory peers, and we explore the reasons for group formation.

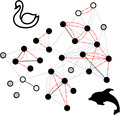

**Abbreviation**
lab: Laboratory

Students' retention, progression and attainment are keenly observed, with seemingly more onus on education providers to ensure positive student outcomes than for previous generations [1]. The students broadly impact the outcomes of these metrics through their behaviour, mindset and interactions with their immediate peer group [2, 3, 4]. An individual's peers provide normative regulation to a group, setting the expected norms and acceptable social behaviours of the group. In the higher education setting, these peer group effects are brought about by the interactions between students within friendship groups, who are often on the same programme of study [5]. Students who find themselves in a group of diligent peers are compelled to work hard and vice versa [6]. Such positive interactions with peers can influence overall academic development, knowledge acquisition, analytical and problem‐solving skills, and self‐esteem [7]. For clarity within this research, peer groups will be defined as follows:

*Social peer group*: the peers an individual interacts with outside of the education setting.
*Education peer group*: the peers an individual interacts with within the education setting.
*Laboratory peer group*: the peers an individual interacts with during laboratory (lab) classes.

There is a wealth of information from primary and secondary education detailing the positive and negative aspects that different types of peer groups can exert on learning [6, 8, 9, 10, 11]. However, within the higher education setting, the dynamics of education peer group formation changes compared to earlier education. Individuals before University form education peer groups based around geographical locations often associated with schools or clubs. On arrival, these students are now in a position to create new groups with individuals from a much wider pool of geographic, cultural and socio‐economic backgrounds. Once formed, a student's immediate education peer group then acts as a reference point for norms during their time in higher education through interpersonal interactions. It has also been recognised for many years that these interpersonal peer groups influence the student's attitudes towards attainment, achievement and further aspirations [12, 13]. This point was directly investigated in a US institution, which showed that students who share rooms with social peers of high academic ability achieve higher grades than those who share a room with students of similar academic ability [11, 14]. Thus, indicating a positive educational influence was brought about by cohabiting with high‐achieving students. This point is echoed by McCabe, who states that students who have friends that provided academic support tended to perform better academically [15]. However, if students do not have friends who motivated them to engage in study, then this can be detrimental to their final attainment [15]. Peer learning can also directly impact an individual's attainment through cooperative activities such as reading each other’s essays and drafts in addition to providing social support [16, 17]. Thus, peer relationships are essential and have been identified as amongst the most influential components in helping students adjust to the demands of learning in higher education and encouraging intellectual self‐confidence [13]. Antonio sets out in his work that students who have best friends with relatively high levels of intellectual self‐confidence ‘tend to be more self‐confident intellectually after two years compared to students with less confident friendship groups’. This indicates that confidence will ‘brush off’ from one social/education peer to another [13]. Racial diversity in the friendship groups was shown to have a positive effect for minority group students on intellectual self‐confidence, suggesting that diverse friendship groups give more varied reference points from which to evaluate themselves and see the inaccuracy of stereotypes [18]. Hence, peer groups and the friendships that students make have a substantial impact on learning, confidence and attainment.

Education peer group interactions are now a staple of active learning in many settings whereby students are encouraged to interact and direct their learning during taught sessions [19, 20]. Within this environment, these groups are either randomly allocated or self‐forming and act to allow self‐directed investigation of a given topic. The groups work to generate outputs or develop their understanding of a topic leading to increased engagement with and understanding of the learning material [20]. Previous research has demonstrated that within the lecture theatre environment, education peer groups predominately form between individuals on the same course even when several modules are shared. These peer groups tend to score similarly on problem‐based tasks where peer interactions are encouraged. Both high‐performing and low‐performing groups were identified on the same course and physically located to the same space in the lecture theatre indicating it is the makeup of the group that is important and not the location in the room or course of study [21]. It was noted by the authors of this study that the students locating together in the lecture theatre were consistent with those students who undertook laboratory practical classes together [21]. Within an alternative laboratory‐based study, students were identified as forming groups with laboratory peers in one of three main categories: peers from a similar cultural background (most importantly sharing the same language), peers with whom they shared their social events, and peers grouping around perceived similar intellectual levels, with the final group gaining good marks [22]. These groups may well be forged at a course or tutor group level with students who progress through similar courses having more opportunities to develop reciprocal relationships with their education peers [23].

To further investigate education peer group dynamics, we wished to determine how and when laboratory peer groups form and the subsequent effect on interaction behaviour and academic attainment. In addition, we wished to investigate whether these groups could be altered to increase attainment of academically weak students. Within the sciences, practical laboratory‐based learning is where students put into practice their knowledge and solve discipline‐specific problems through collaborative learning. Students tend to spend a large proportion of their contact time within the laboratory class setting and work closely with each other through the sessions. Laboratory groups can range in size from 20 to 100 depending on the institution; students generally work with a laboratory partner for the duration of a class who they are free to select based on the pool of individuals who have elected to take the given course. As the laboratory group setting sees students working intensely with their peers, this study starts with how students choose their laboratory partner(s). The study then focuses on the resulting laboratory class group formation networks, the influence these have on students–peer interactions and attainment and the effect of an intervention to widen the students' laboratory and education peer groups.

## Methods and ethics

### Participants

In the first year of the study, the student participants were first year cohorts on a range of Biosciences and Chemistry undergraduate degree programmes comprising of Chemistry, Biomedical Science, Biochemistry, Biology and Human Biology. Within all the course cohorts investigated here, the practical laboratory experience is central to the curriculum with theoretical aspects of the courses being put into practice in this learning environment. For the first year of the study, from a total cohort of 266 students, 193 students were polled and opted into the study. In the second year of the study, the student participants were first year cohorts on Biochemistry, Biology and Human Biology undergraduate degree programmes, with 83 students opting into the study from a cohort of 100. On all degree programmes in question, students take part in day‐long practical sessions, occurring weekly, with courses split into groups of 25–30 students. These classes account for approximately 35% of the student's total contact time (which also includes lectures, tutorials and workshops). Laboratory sessions are a mandatory component of the degree programmes, with a 70% pass/fail attendance criteria. Students are free to work with whomever they like within the laboratory group and typically work in pairs during a given class and are free to change whom they work with on a week‐to‐week basis.

### Collection of data

Students were surveyed within the last three laboratory sessions of the core laboratory module in the first year of study, using a printed questionnaire during normal timetabled laboratory classes. These questionnaires asked students to identify who their laboratory partner had been within that year of study. The students were then asked through a selection of preformed responses to think about each student they had partnered with and identify how often they had worked with them and why they choose to work with them. An open text question was also included to allow the students to elaborate on why they decided to work with a given individual. Finally, the students were asked to identify how strongly they associated with a range of statements around course identity and study habits on a Likert scale of 1–5.

### Ethics

Ethics for this study was acquired through the College of Health, Wellbeing and Life Sciences ethics committee following the Sheffield Hallam University Research Ethics Policy. Ethical approval was given as no identifiable, confidential or controversial information would be collected. No gender, age, other educational experience or other demographic factors were requested or considered within the analysis, primarily to ensure the questionnaire was concise and the length not a barrier to completion. Participation in the study was optional, and the students were read and given a copy of the following statement before the collection of data, which served as a means of consent.

Your name and student number will not be used in any publications. If you do not wish to take part in this study, it will not affect your grades for this module. If you are unclear about anything or have further questions, please feel free to ask or email m.lacey@shu.ac.uk.

If you wish to opt‐out of the study, simply do not hand in your completed questionnaire. Submission of completed forms will be accepted as your consent to participate. After submission, you will not be able to withdraw from this research study.

### Data analysis

The student questionnaires were collected, and an independent researcher indexed and transposed the details and comments into Excel. Students' names in the study were replaced with their student number to prevent bias by the investigator and link the responses to attainment. Students self‐identified the laboratory peers they had worked with as ‘always’, ‘most of the time’, ‘occasionally’ or ‘once’ in previous laboratory classes, and these interactions were visualised as a complex network using Cytoscape_3.6.1. Briefly, responses were processed for further analysis by determining for each student, the student number for each individual they have worked with and the level of interaction. Course group was uploaded individually into Cytoscape_3.6.1 as a ‘galFiltered’ network [24]. Types of interaction (always, most of the time, occasionally or once) were visualised by different line styles. Models were exported as a.png file, and the type of group was superimposed. Students were put into groups based on which students they identified they had worked with ‘always’ and ‘most of the time’. Groups containing two students who work exclusively with one another ‘always’ and ‘most of the time’ were labelled as ‘swans’ and groups with three or more students were labelled as ‘dolphins’. Students who had no fixed group were excluded from the main element of the study due to their low number. The average overall first year module mark for all the students within a group was recorded. Those students within groups with the higher average marks were said to be in a high‐attainment group and those in a group with lower than average marks were said to be in a low‐attainment group. This was undertaken separately for swans and dolphins. Correlations between the class average first year mark and individual first year mark were undertaken for the identified group types. How students indicated they met their laboratory partner(s) was tallied for those identified as ‘low‐attainment swan’, ‘high‐attainment swan’, ‘low‐attainment dolphin’ and ‘high‐attainment dolphin’. Using a Likert scale, the same criteria were used to evaluate the responses to investigate course identity and study habits.

### Statistics

Comparison of individual group median scores was performed with the program StatsDirect. In order to maintain student anonymity, attainment data were plotted as the difference to the overall class mean of the students' end of year average. Median ranked final group marks for dolphin groups were analysed by a Kruskal–Wallis: all pairwise comparison followed by a post hoc Conover–Iman test. Statistical significance is indicated by asterisks throughout the manuscript; * indicates *P* < 0.05. Correlation between individual student marks within the swan pair groups was determined by Pearson's correlation coefficient.

Responses to Likert scale questions were converted to numbers from 1 to 5 for statistical analysis, with 1 representing the most negative response to the question (e.g. strongly disagree) and 5 the most positive (e.g. strongly agree). The ordinal nature of the questionnaire data meant that parametric statistics were inappropriate for analysis, so nonparametric tests are used throughout. Observations were independent, with no individuals belonging to more than one study group. For comparisons of multiple groups, the Kruskal–Wallis *H*‐tests were used, followed by pairwise post hoc Mann–Whitney *U*‐tests between particular groups where appropriate. Statistical significance is indicated by asterisks throughout the manuscript; * indicates *P* < 0.05.

## Results

This study set out to determine the effect of laboratory peer group formation on attainment and those factors driving friendship group formation within the first year of a UK‐based Biosciences and Chemistry degree programme of study. Students were asked to identify the people they worked with during laboratory‐based practical classes and the frequency that they worked with them. Data were collected during the second semester of the first year of study through a paper‐based questionnaire. The study was opt‐in with completion of the questionnaire acting as consent to the study. A confidential statement was also included on the form and being repeated orally before data collection. Of the 266 possible responses for the 2017/18 cohort, 193 were received (73% response rate). Full ethical consideration was given to the questionnaire and the handling of the data through the standard University‐wide procedures. Student ID numbers were collected, which allowed mapping to end of year final attainment.

### Cluster analysis—swans and dolphins

To identify the laboratory peer group networks, we asked the following question:

Please list all the students you have worked within the lab up to this point. Thinking about each student, tick how often you have worked with them in the lab this semester.

Responses were recorded on a tick sheet as ‘*always’*, ‘*most of the time’*, ‘*occasionally’* or ‘*once’*. A cluster analysis using Cytoscape_3.6.1 was performed on the resulting data sets to visualise the types of complex interactions. A strong connection was assumed if the students responded ‘*always’* or ‘*most of the time’*. Responses were cross‐referenced and verified to ensure all students in a cluster identified the other members as being laboratory partners either ‘*always’* or ‘*most of the time’*. The data sets revealed two common cluster types:

#### Swans

Long‐lived well‐established pairings. Two students would exclusively identify each other as laboratory partners ‘always’ or to a lesser extent ‘most of the time’ and would work together for all or the majority of the laboratory sessions during the full duration of the year. Interactions with other students outside of the pair would be identified as ‘occasionally’ or ‘*once’*.

#### Dolphins

Fluid groups of students that work interchangeably. This group of students was typically 3 to 5 (range 3–9) in size with a mode of 4 containing members that identified other students as partners ‘most of the time’. Which students paired in a given laboratory session was dynamic within the group.

A third and much rarer student type was also identified, with only two observed in the initial data collection.

#### Albatrosses

Students who identified as only having partners that they worked with ‘occasionally’ or ‘once’. Due to the very low number of students within this category, they were excluded from the main elements of the study.

**Fig. 1:**
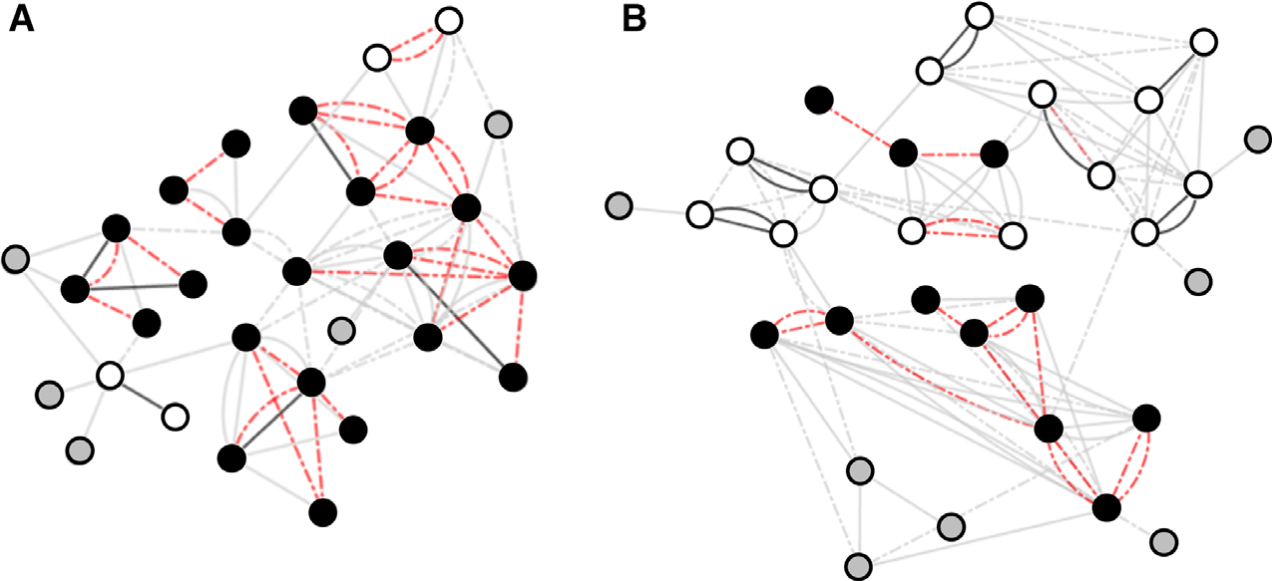
Complex network analysis of student laboratory partner interaction in the first year of study in (A) Biochemistry and (B) Biology degree programmes. Circles denote students and lines the type of interaction with the laboratory classes, students working together always (solid black line), most of the time (dashed red line), occasionally (solid grey line) or once (dashed grey line). White circles indicate swans, and black circles indicate dolphins. Grey circles indicate an albatross or a student not in the study mentioned by another.

Different courses had different proportions of long‐lived swan pairs and dynamic dolphin groupings (Fig. 1). Biology, for example, was typified by swan pairings (with 16 from 22 individuals identifying as swans), whereas Biochemistry was mostly dolphins (with 18 from 26 identified as working in this group type) (Table 1). Notably, two swan groups would identify each other as working together ‘some of the time’, indicating that on occasion, the pairings would break allowing a third student to join. Inclusion of a third member presumably occurs when one member of a swan group was unable to attend class and temporarily joined another pairing. Swan pairings reported only a handful of their laboratory peers that they worked with ‘occasionally’ or ‘once’ suggesting they would exclusively work together for the whole academic year.

**Table 1:** Summary group data indicating the number of students on each course who were identified as either swans pairs or dolphins groups. Albatrosses were excluded from the main elements of the study due to low numbers.

Course	Number of students identifying as swans	Number of swan pairings	Number of students identifying as dolphins	Number of dolphin pods
Biochemistry	8	4	18	3
Biology	16	8	6	1
Biomedical Science	38	19	36	9
Chemistry	34	17	5	1
Human Biology	2	1	9	3
Totals	98	49	93	24

### Attainment—swans and dolphins groups have similar levels of attainment

**Fig. 2:**
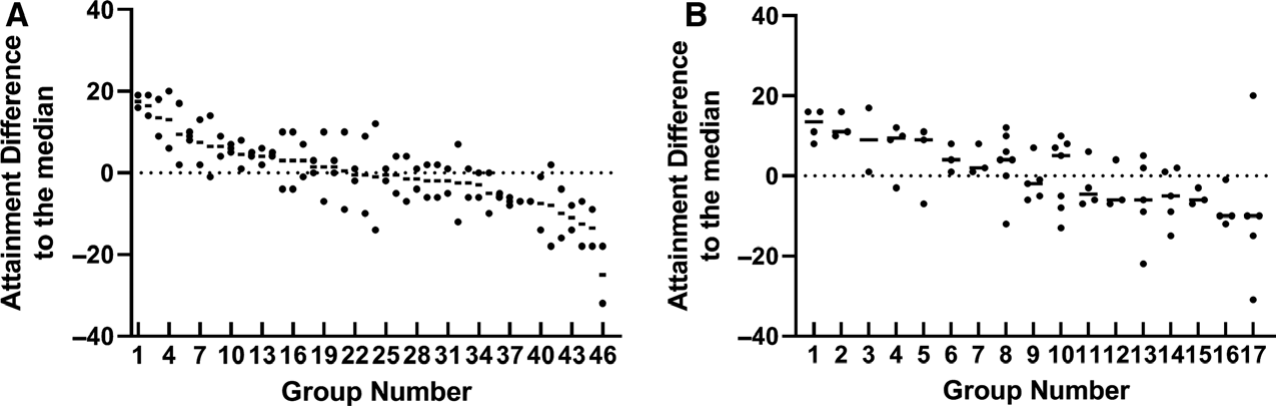
End of year attainment grouping of (A) swan pairs or (B) dolphin groups from the 2017/18 cohort. Student anonymity was maintained by plotting the data as the difference to the overall class mean and individual groups ranked from high to low attainment. Each point represents an individual student's mark within a given group.

Having identified that laboratory peers form discrete groups during laboratory practical taught sessions, we set out to determine whether students within these groups had similar levels of attainment to each other. To do this, attainment within each group was compared to other groups by analysing the end of year average mark (Fig. 2). The data were arranged by taking the median mark of the individual group and ranking these from highest to lowest. The ranked data are presented as the difference to the class median to preserve the anonymity of the students. There was no statistical difference in the average attainment across courses with no one course outperforming another (data not shown). What is remarkable in both the swans and dolphins data sets is how closely the attainment of members of a given group was (Fig. 2). Pairwise comparisons between the two students in a swan group gave a Pearson's correlation coefficient value of *R* = 0.3867, with a *P* = 0.008 from the 46 pairings. If one member of a pairing performed well, then it was likely that the other member would also perform well. The attainment of the dolphin groupings was analysed by a Kruskal–Wallis: all pairwise comparison (Initial *P* = 0.0183) followed by a post hoc Conover–Iman test. Table 2 shows *P* values gained from this analysis. Students appear to cluster in groups of similar abilities with high‐performing students performing significantly better than groups of low‐performing students. Hence, students who identified as working together during laboratory practicals obtained similar levels of attainment in assessments on the course in general.

**Table 2:**
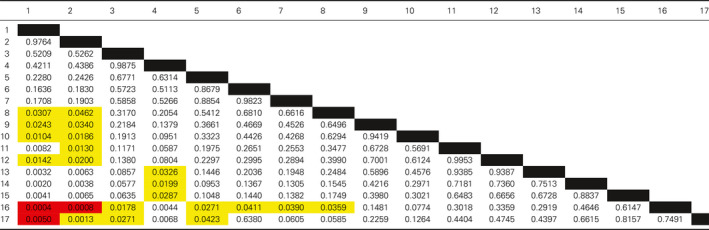
Significance testing between dolphin groups was determined by Kruskal–Wallis all pairwise comparison followed by a post hoc Conover–Iman test. The initial *P* value of the test was 0.0183. Significance between individual groups was reported with a *P* value of > 0.05 and is indicated on the figure by yellow > 0.05, orange > 0.01 and red > 0.005.

### High‐attaining students value a similar work ethic over background when choosing laboratory partners

Having identified that students form stable groupings and that members of these groups have similar levels of final attainment, we next investigated the rationale for their formation. Akhtar reports that the drivers for peer group formation are a sharing of social events, perceived similar intellectual ability and shared cultural background, most importantly sharing the same language [22]. Within the study performed here, students were asked to identify the reason they were working with other individual students by identifying one or more of the following statements:

- ‘I knew them before I started university’
- ‘We met in during induction week outside of the department’
- ‘We met on the course we have a similar appearance’
- ‘We met on the course and we have similar background’
- ‘We met on the course and we have a similar level of engagement’
- ‘We met on the course and we have a similar academic attainment’

**Fig. 3:**
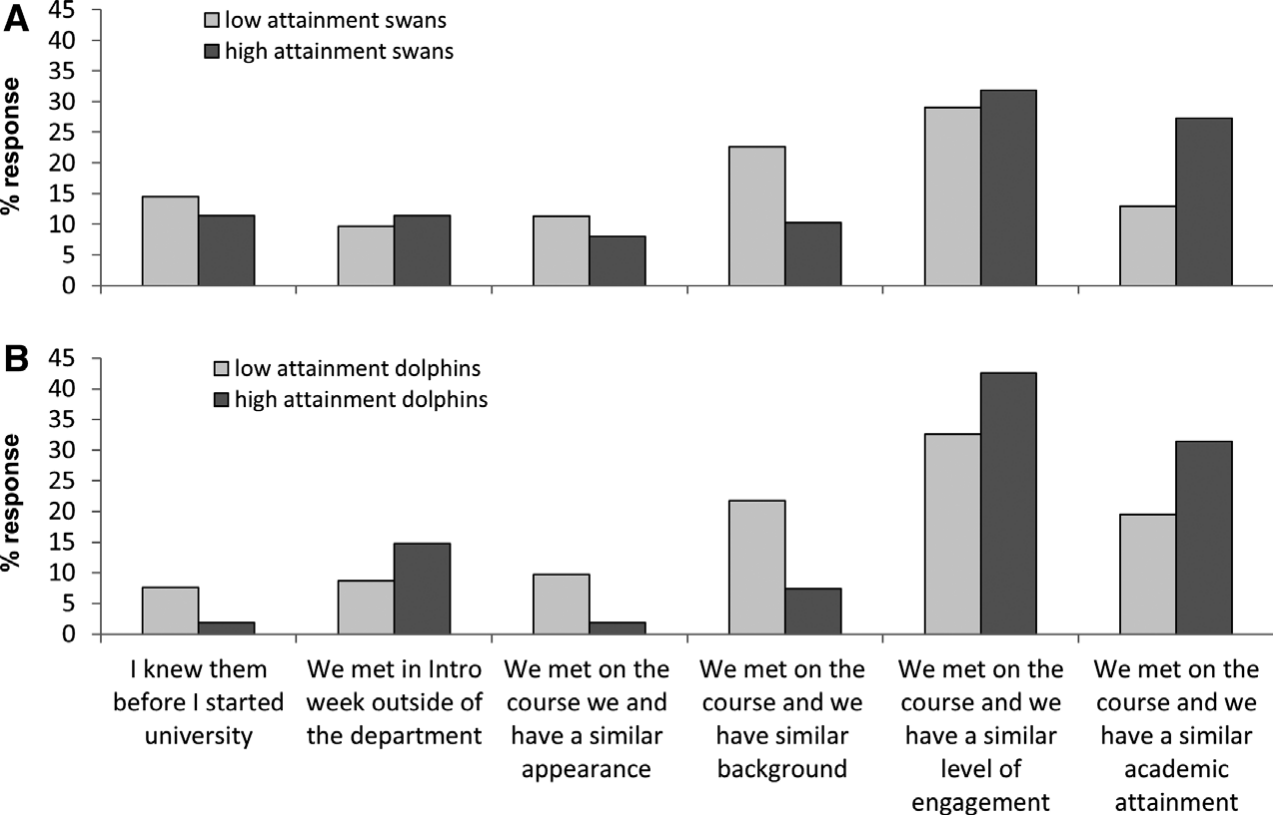
Percentage response to laboratory peer group formation questions from (A) swans pairings and (B) dolphin groups. Students were asked to identify the reason they were working with other individual students highlighting one or more of the above statements. The responses were collated and the data split between those students performing above the class median (high attainment) and those students performing below it (low attainment). Data were recorded for individual students who they worked with ‘always’ or ‘most of the time’.

The responses for the swan and dolphin groups were collated and the data split between those students performing above the class median (high attainment) and those students performing below it (low attainment) (Fig. 3). For both the high and low long‐lived pairing swan and dynamic dolphin groups, the main reason given for group formation was a perceived similar level of engagement. Within the swan groupings, the majority of response rates between the high and low groups were the same; however, two questions stood out and have deep implications for inclusion and practice. Swan pairings in the high‐attainment group indicated that they work together due to a similar level of academic attainment, 26% of the time as opposed to 13% in the low‐attainment group. Conversely, students in low‐attainment group indicated that they worked together because they identified as having a similar background, 23% of the time as opposed to 10% of the time for the high‐attainment group. The pattern of responses was broadly repeated with the dolphin groups in that the high‐attaining group put a higher value on engagement and attainment. The low‐attainment group, however, valued laboratory peers with a similar background or appearance. This would indicate that high‐performing students are seeking out laboratory peers with a similar academic outlook and work ethic. In contrast, lower‐performing laboratory peers put more value on a similar background and perceived social group.

Further to this, an open‐text response question was included to allow the students to indicate why they were working with a given individual. One hundred and sixteen responses were gained from this question. Quotes were gathered at the midpoint of semester two and thus represent the views of the students once the laboratory peer group had been established. Analysis of the responses for each quote was blinded and then coded into themes. Response fell into three main categories:

1. Good working relationships (42 responses):
  ‘Similar level of engagement’
  ‘Have a similar way of thinking’
  ‘We always worked well together’
  ‘We get on well and can work together as we both listen and share ideas’
  ‘Similar engagement and reliable’
  ‘Comfortable working together’
2. Friends in a social capacity (31 responses):
  ‘Friends outside labs’
  ‘We get on well and most people on the course aren't approachable’
  ‘Good mate, work well, good laugh’
  ‘Family friend’
  ‘I knew her before I started university and I feel very comfortable working with her’
3. Friends/Good working relationships (18 responses):
  ‘We are good friends and work well together’
  ‘We work better together, good chemistry and socialise outside of labs’
  ‘They are good friends of mine in and out of the labs. Hardworking and also take their work seriously’

There was no clear split in the open text responses between the different group types or attainment levels. When students are allowed to choose their laboratory partners, it appears that a similar work ethic and friendship bonds are the basis behind laboratory group pairings.

### Implications for working practices

**Fig. 4:**
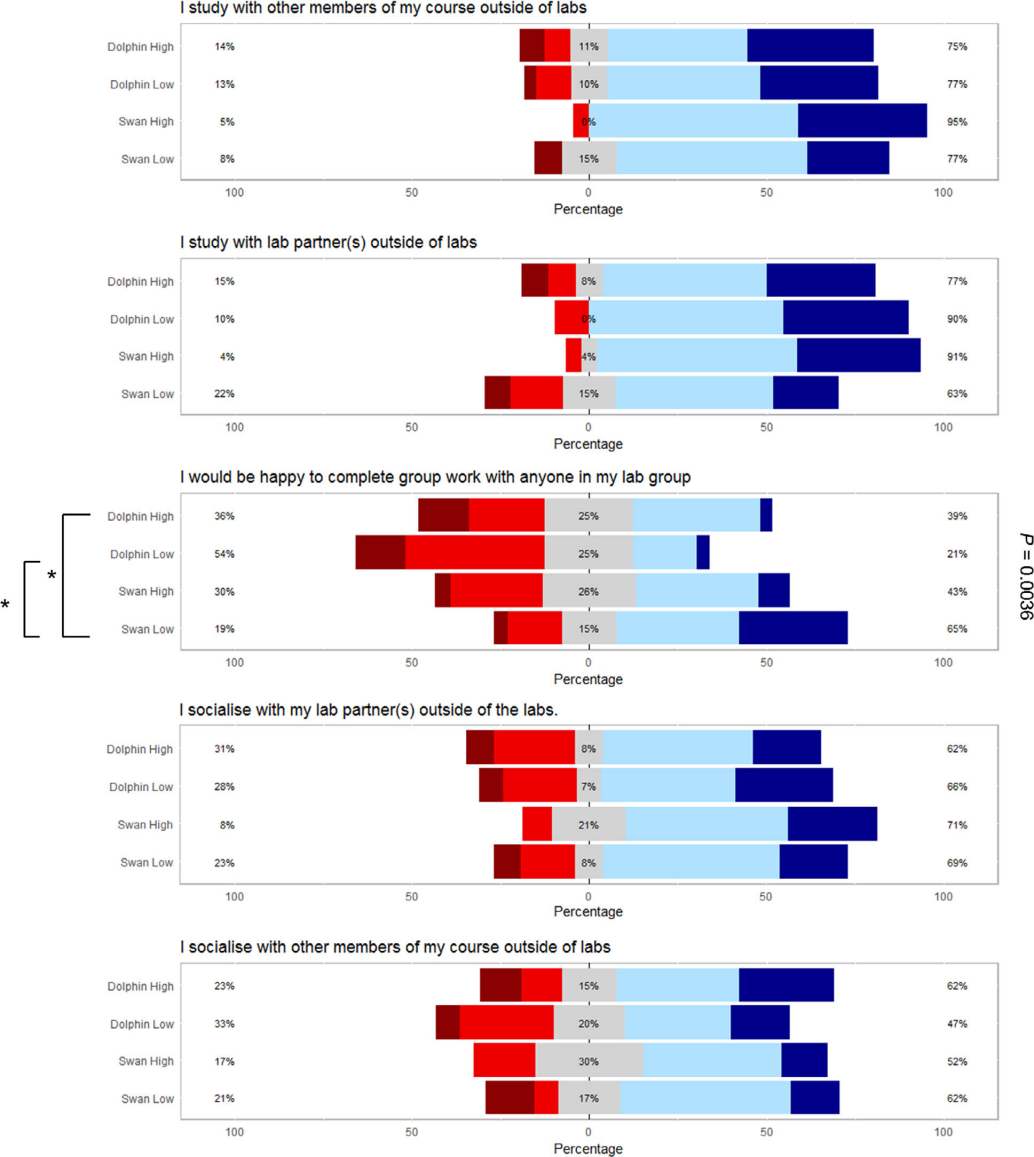
Likert scale data obtained into student interaction behaviour. Dark red = strongly disagree, red = disagree, grey = neutral, blue = agree, dark blue = strongly agree. Lines and quoted *P* values represent Kruskal–Wallis test for differences between the four types of laboratory peer groups. Asterisks represent all pairwise comparisons (Conover–Iman) post hoc tests for differences between pairs of laboratory peer groups. For initial tests of difference, * represents significance at α = 0.05; for post hoc tests, * represents significance at *P* < 0.05.

Having determined the basis by which students form their individual laboratory groups, we wanted to understand the implications on educational and social interactions. Students were asked a series of questions relating to course identity, education peer group and their working practices. Responses were rated as on a Likert scale between 1 (strongly disagree) and 5 (strongly agree). The responses were collated and the data split between the two group types and those students performing above the class median (high‐attainment groups) and those students performing below it (low‐attainment groups) (Fig. 4). Approximately 80% of students recorded that they either agreed or strongly agreed that they study with their laboratory partner outside of the laboratory irrespective of group. Thus, a student's laboratory peer group is most often synonymous with their education peer group. In addition, students also predominantly agreed that they socialise with their laboratory group and are thus also social peers.

Notably, but not statistically verified, a lower proportion of the low‐attainment swan group agreed with the statement that they study with their laboratory partner (63% of respondents). This would mean that the students are either working alone or with someone who is not their laboratory partner. In support of this hypothesis, a significantly higher proportion of the swan low‐attainment pairings agree with the statement, ‘I am happy to complete group work with anyone in their lab group’ (*P* > 0.05). A willingness to study with others may well then explain the increased variability in final attainment seen between the pairings in the lower attainment swan group (Fig. 2).

The observation that laboratory peer groups are studying together and thus are also education peer groups may well help explain why laboratory peer groups obtain similar overall marks at the end of the year assessment (Fig. 2). This also allows the responses as to how students chose their laboratory partner(s) to be viewed on a course level for education peers, and as such, these data can inform policies and interventions in non‐laboratory‐based educational environments.

### Intervention: initial group formation by random allocation as opposed to self‐selection

Students on the degree programmes within this study undertake four, 1‐day laboratory, practical classes, over four weeks at the start of their studies as a means of orientation. In the data set above, the students were allowed to self‐select their laboratory partners within these induction sessions. We hypothesised that by allocating students to work with different laboratory partners over this induction period, we would be able to facilitate introductions and bring about a broader sharing of knowledge and experience. Students on the Biology, Human Biology and Biochemistry courses were randomly allocated a different laboratory partner in each of the four practical sessions during the induction period. They were informed at the start of the first laboratory session who they would be working with on that day and for the subsequent 3 weeks. For reasons of resources and practicality, students studying on Biomedical Science and Chemistry courses were not included in the intervention. The questionnaire was repeated with the Biology, Human Biology and Biochemistry of this intake during the second semester of the first year of study through a paper‐based questionnaire to mirror the timing of the first study. From the 100 students within the 2018/19 cohort, 83 students were polled and opted into the study (83% response rate). Cluster analysis was again performed on the students who had received the intervention and compared to the Biology, Human Biology and Biochemistry students’ clusters from the original 2017/18 study cohort (Table 3). A two‐sided Fisher's exact test demonstrated a significant change in the distribution between the groups (*P* = 0.004) with a higher proportion of students identifying as dynamic dolphins following the intervention.

**Table 3:** Summary group data indicating the number of students on in each cohort who identified as either swans pairs or dolphins groups. Albatrosses were excluded from the main elements of the study due to low numbers.

Cohort (Biology, Human Biology, Biochemistry)	Number of students identifying as swans	Number of swan pairings	Number of students identifying as dolphins	Number of dolphin pods
2017/18	28 (67%)	14	33 (34%)	8
2018/19	14 (33%)	7	65 (66%)	16

Within the 2018/19 study, the participants identified 484 students they interacted with. Of these interactions, 7% of laboratory peers were identified as worked with always, 24% most of the time, 29% occasionally and 40% once. Of students where the participants were randomly paired with in the first four laboratory sessions (161 interactions), 1% of laboratory peers were identified as worked with always, 11% most of the time, 19% occasionally and 70% once. This indicates that the students were not forming long‐lasting relationships with those laboratory peers they were randomly allocated to, but instead seeking out laboratory peer groups independently.

### Following the intervention, a comparison of the final attainments of each of the group types shows no change in grade distribution

**Fig. 5:**
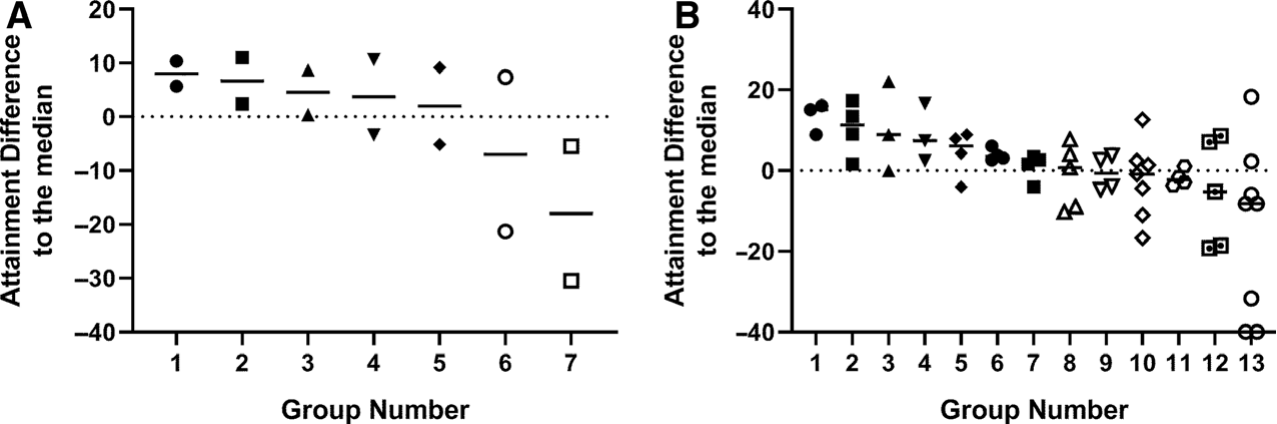
End of year attainment grouping of (A) swan pairs or (B) dolphin groups from the intervention (2018/19) cohort. To maintain student anonymity regarding the final mark, acquired data had been plotted as a difference to the overall class mean and individual groups organised from high to low attainment. Each point represents an individual student's mark.

The average end of year attainment for the intervention and the previous year's students was very similar with no statistical difference (58.9 ± 11.1% vs. 57.5 ± 11.1% respectively). The intervention cohort end of year attainment was subsequently analysed in the same manner as with the previous year's students (Fig. 5). A Kruskal–Wallis: all pairwise comparison was performed (initial *P* = 0.0224) followed by a post hoc Conover–Iman test. Table 4 below shows *P* values gained from the pairwise analysis. The pattern of attainment matched that seen for the 2017/18 cohort with distinct high‐performing groups doing significantly better than low‐performing groups. Due to the limited number of swan pairings and high variability in the low quartile group, a positive correlation between the swan end of year marks could not be determined. However, a pairwise comparison of the combined data from the 2017/18 and 2018/19 cohorts did return a Pearson's correlation coefficient value of *R* = 0.3137, with a *P* = 0.019 from the 55 swan pairings. This correlation coefficient identifies a modest but significant positive correlation between the end of year marks of the two years of the study when combined with the total data set.

**Table 4:**
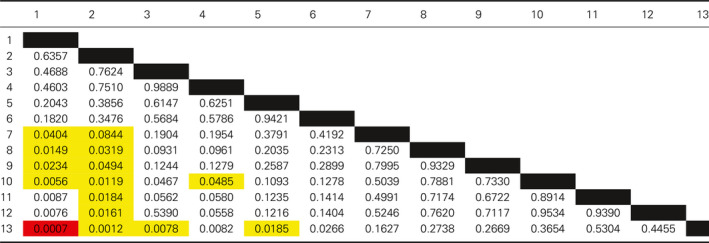
Significance testing between dolphin groups in the 2018/19 cohort was determined by Kruskal–Wallis all pairwise comparison followed by a post hoc Conover–Iman test. The initial *P* value of the test was 0.022. Significance between individual groups was reported with a *P* value of > 0.05 and is indicated on the figure by yellow *P* > 0.05, orange *P* > 0.01 and red *P* > 0.005.

Within the study, only five students were identified as albatrosses, two in the first year and three after the intervention. The average end of year attainment of these albatrosses was 60.5 ± 8.2% compared to the whole study average of 58.5 ± 11.1%. It is important to note that these albatrosses are likely not representative of all students that would be identified as albatrosses. Within each student cohort, there is a subgroup of students that disengage from the laboratory module. These students do not have a weekly laboratory partner and would most likely be identified as albatrosses, but, as they were not part of the sample of student responses collected, they fall out of the research focus and ethical framework of this study.

### The intervention increased student's willingness to work with others

**Fig. 6:**
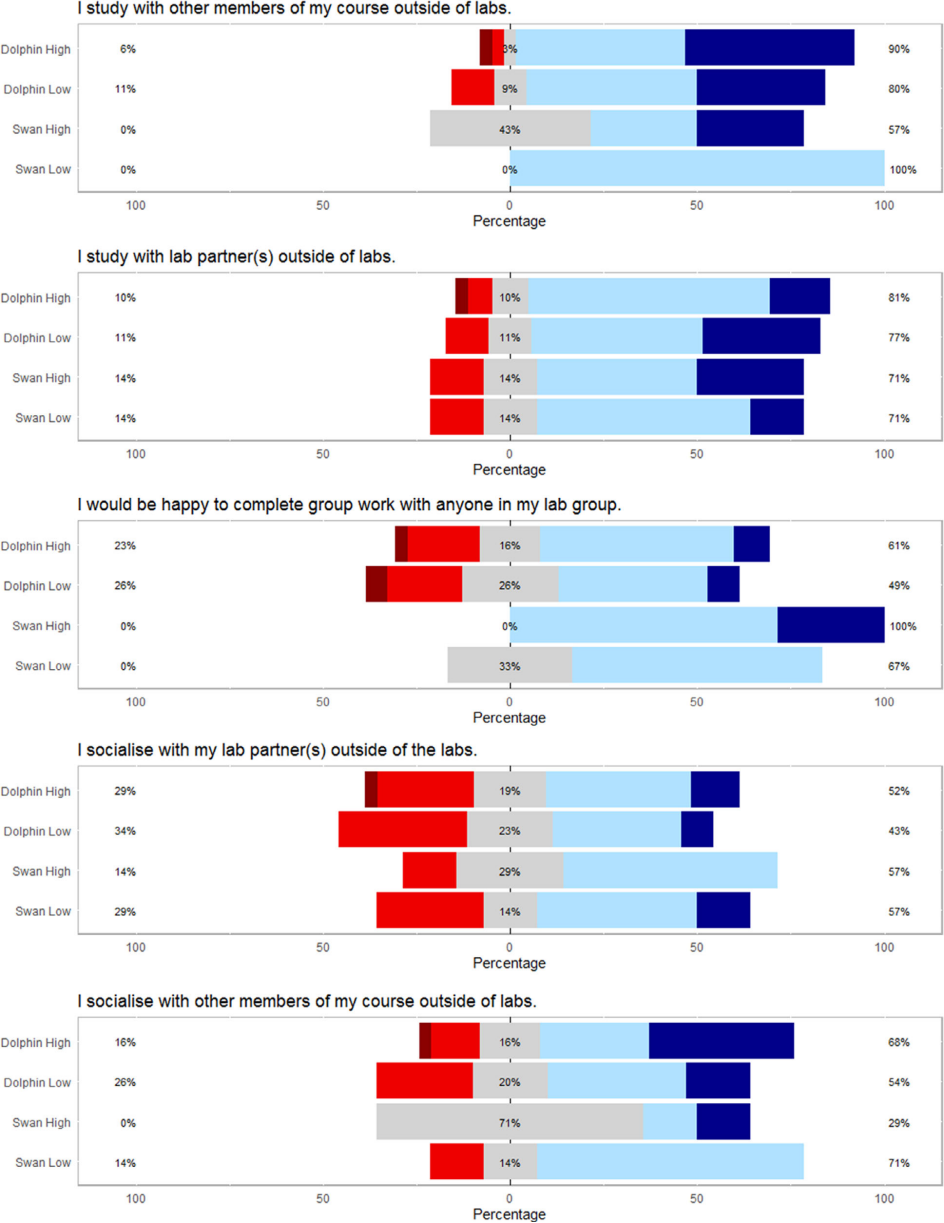
Likert scale data obtained from student interaction behaviour from the 2018/19 cohort postintervention. Dark red = strongly disagree, red = disagree, grey = neutral, blue = agree, dark blue = strongly agree. Lines—no significant differences between the group responses were not observed following Kruskal–Wallis analysis.

**Fig. 7:**
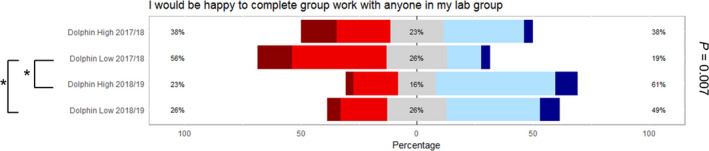
Likert scale data obtained from the 2017/18 and 2018/19 student cohort into their interaction behaviour. Dark red = strongly disagree, red = disagree, grey = neutral, blue = agree, dark blue = strongly agree. Lines and quoted *P* values represent Kruskal–Wallis test for differences between the four types of laboratory peer groups. Asterisks represent all pairwise comparisons (Conover–Iman) post hoc tests for differences between pairs of laboratory peer groups. For initial tests of difference, * represents significance at α = 0.05; for post hoc tests, * represents significance at *P* < 0.05.

The intervention students were asked the same series of questions relating to course identity and their own working practices as the previous year's students (Fig. 6). In all groups, students predominantly agree with the statements that they study and socialise with their laboratory partner. In this way, it can be inferred that interactions are maintained outside of the laboratory in a social and academic context. The responses were not significantly different from the original cohort apart from the response to the question ‘I would be happy to complete group work with anyone in my lab group’. In this case, attitudes had significantly shifted within the dolphin group from predominantly disagreeing with this statement to agreeing (Fig. 7). Together, these data indicate that by facilitating the interaction between different students during the induction period, a higher proportion of students are willing to pick laboratory partners from a wider group and being more willing to undertake group work with other students on their course. This has the potential implication that in future group work scenarios, those students involved may be more willing to conduct short‐lived tasks with peers outside of their immediate peer groupings.

## Discussion

This study aimed to investigate laboratory peer group dynamics within a laboratory environment, to understand how peer groups form, the drivers for peer group formation and the effect of these groups on attainment and laboratory and education peer interactions. The main intervention within the study aimed to determine the effect of self‐selecting vs. randomly allocated initial student interactions and the subsequent effect on attainment and laboratory and education peer group interactions. Initial analysis of the group dynamic of student laboratory partners identified two distinct group types named here as ‘swans’ for students predominantly working in pairs and ‘dolphins’ for more dynamic student groups of three to nine members. We also identified within the data sets a third group, ‘albatrosses’, with a low number of students who did not associate with either of the two group types and appeared to join others for laboratory practicals on an *ad hoc* basis. This level of analysis for peer group interaction within a teaching environment is novel but is supported by the literature as peer group dynamics have also been identified for students' entire friendship groups (social and education peers) in a study by McCabe, following structured interviews with a range (socio‐economic group and ethnicity) of students in the United States throughout their college programme [15]. Three different types of student groups are reported; tight‐knitters, who generally stick to one group, compartmentalisers, who have two to four clusters of friends, and samplers, who tend to have friends that do not know each other as they are ‘*sampling’* friendships. Within these studies, the group of friends who meet on a course either merge with those from home (tight‐knitters), or remains discrete (compartmentalisers and samplers), no further resolution into the type of friendships on a course has been sought. Berthelon (2019) shows clusters of students within study groups in Chile in the 4^th^ and 5^th^ years of study, these groups have variation in the amount of connectivity similar to that reported here [17]. Our work shows that student groups which formed at a module and course level can be categorised into types of groups, which are similar to those across all of a student's friendships. As such observations were also seen by McCabe, these types of group formation are seemly ubiquitous amongst student populations.

The effect of education peer group on academic attainment and engagement has been greatly discussed within the literature. A large proportion of this literature on peer effects in HE is drawn from US institutions and often sampled from situations where room‐mates are randomly allocated. Classroom peers (education peers) and college room‐mates (social peers) are known to improve educational achievement [6, 8, 9, 10, 11]; however, in these studies only modest sized effects from ‘dormmate’ and ‘room‐mate’ background on a student's academic performance are observed [25]. Our study supports this literature by showing the formation of high‐ and low‐attainment groups, with group members gaining similar grades, especially within the high‐attainment groups [3, 10, 11, 17]. Compounding effects within the high‐attainment group on the individual’s academic performance occurs presumably due to education and social interactions positively affecting the academic performance of the individual [6, 9, 11]. It would be tempting to speculate that by intervening and randomising students, those who would have been in the lower attainment groups could overcome initial friendship barriers and work with high‐attaining peers to improve grades. After our initial study in which laboratory and education peer groups were self‐forming, an intervention was undertaken by allocating random laboratory partners for the first four laboratory sessions. We observed a shift from long‐lived swan pairings to dynamic dolphin groups between the years but can only infer this was due to the intervention rather than a change in the cohort.

Within our study, laboratory peer groups and education peer groups greatly overlap and there was no observable difference between the two laboratory peer group types (swans or dolphins) in terms of final attainment, indicating that it was not the size or the dynamic of the group that mattered, more the members within the laboratory/ education peer groups. The attainment data taken from the end of year average mark of both the initial and intervention groups demonstrated that members of individual groupings tended to score similarly regardless of the peer group type. Within our study, the students have selected to study the same course and work with peers with similar interests and either agreed or strongly agreed that they study and socialised with their laboratory partner outside of the laboratory. This observation that laboratory peer groups also form education peer groups may well help explain why they obtain similar overall marks at the end of the year assessment. The fact that mark clustering in course groups aligns with friendship groups has also been reported in our past investigations into lecture seating [21]. In that environment, course‐based friendship groupings located together in the lecture theatres, with clusters of high‐ and low‐performing education peer groups obtaining similar marks on problem‐solving tasks. Sacerdote, within their review, noted that high‐ability students benefit from the presence of other high‐ability students [4, 8].

The choices students make around whom they associate with can have a dramatic influence on their University experience. A significant positive relationship between the quality of new friendships and adjustment to University has been shown [26]. The association between the two was stronger for students living in residence than for those commuting to University, due to the need to make new friends rather than accessing established networks [26]. The process of making new friends in the Buote [26] study was reported to start as soon as they arrived and is echoed in the data presented here, with very few students stating that they knew each other before starting the course. Group formation then occurs quickly once on the course and is based on locating and interacting with peers with a similar academic outlook and/or social grouping. There appears to have been little movement throughout the year with the group, although the intervention appears to increase a student's peer network, and thus, they are more likely to be a dynamic dolphin, where members of these pods are formed early in the year and are not based on whether the students met during the intervention. Thematic analysis of the free‐text comments also gave context and highlighted two main themes in the way the peer groups were forming, with good working relationships and being friends in a social capacity emerging as strong reasons for group formation. Although there was no difference between the ‘dolphin’ and ‘swan’ groupings in this study around the reasons for group choice, there was a difference between high‐ and low‐attaining students. High‐attaining students tend to form laboratory/education peer groups around ability and work ethic, whereas low‐attaining students form laboratory/education peer groups around perceived similar background and appearance.

Friendship groups have been reported to provided tangible assistance in work and role models of appropriate behaviour [26] with the academic quality of a study group (education peer group) having a strong effect [17]. It has also reported that students are more likely to form study relationships the more aligned they are along several dimensions, such as ability, socio‐economic background, age and geographic origin, amongst others [17]. In a laboratory‐based computer‐aided design, correlation analysis between learning outcomes and student interactions revealed that students who sit in groups have higher average scores. The results show that student achievement was positively correlated with attendance, social stability in terms of laboratory peer grouping, and time spent on task [22]. Laboratory peer groups formed on the basis of perceived similar intellect also appeared to gain good marks [22], and high‐ability students have been shown to gain more from interactions with other higher‐ability peers [27]. Manipulation of peer groups to optimise the learning gain for low‐ability students can have negative consequences with students avoiding interaction and forming homogeneous subgroups [28]. In this work, although good working relationship and similar work ethics were cited as reasons for peer group formation, this was seen for both the high and low groups. We cannot ascertain from the data whether the laboratory/education peer group formation is because the students involved are equally willing to place an effort into study and learning or more willing to coast at a similar rate. Notably, the low‐attaining peer groups were forming more around concepts of familiarity, likely leading to a lack of social mobility. Here, intervening to enable students to work with different pairings during the early part of the semester did not lead to a change in attainment. It was noted that these random pairings did not lead to long‐lasting working relationships as most students reported only working with their randomly allocated partner once (70%) or occasionally (19%). Hence, it was speculated that students likely formed strong peer groups with the same or similar students as they would have done without the intervention. The average end of year attainment for the pre‐ and postintervention groups was the same, as was the spread of marks. Interestingly, although no difference in end of year assessment was observed, students were more likely to work with a wider pool of peers during taught practical sessions representing a softer change in the overall groups dynamic.

The research undertaken here reinforces the impact of peer groups and the difficulty in altering a student's grade by attempting to alter their education peer group and the group dynamics within the laboratory (and education) environment. Further research into how the laboratory/education peer groups described here alter through a degree programme would be of interest as would more detailed analysis of students' demographics and their effects on education/peer group formation. Insight into the chicken and egg conundrum whether student attainment is affected by a student's education peers group, or whether students form education peer groups with those likely to achieve similar attainment would be of great value to allow further attempts to increase the attainment of low‐attainment groups.

### Implications

Peer‐to‐peer interactions and collaborative learning are central themes in higher education. Collaborative learning is a situation in which two or more people learn or attempt to learn something together [20] and how students work collaboratively greatly affects their time in higher education [17]. This study, and the discussion of the wider literature, leads several implications.

#### Like begets like

Academics should be aware that students of similar ability are forming long‐lived networks with each other. Students in the low‐attainment education peer groups are substantially more likely to form a group with those with similar backgrounds and appearance as opposed to those in high‐attainment education peer group, which form around perceived effort. This has implications for initiatives based around widening participation and inclusion.

#### Students gain from group learning

Unlike individual learning, students engaged in collaborative learning capitalise on one another's resources and skills [19, 20]. Interpersonal relationships established in the laboratory set out the study and support groups the student will later work within. This collaborative learning has been demonstrated to: enhance problem‐solving skills, inspire critical thinking, improve social interaction, support diversity, and aid the development of self‐management and oral communication skills [20, 29, 30]. Students attaining lower marks are likely to be found in either swan or dolphin groups and initiatives to widen a student's network, such as those seen here, affect the group dynamic but not the overall attainment. How academics can best support those in the lower attainment peer groups regardless of their formation is then a critical factor for improving retention, progression and achievement.

#### Groups are hard to change

Interventions such as random allocation during group work will not be the primary catalyst for long‐term friendship but will enable students to be more willing to work with others in future tasks. Academics should focus on utilising short transient mixed ability groups to support lower attaining individuals rather than attempting to alter group dynamics at a module or course level. Transient support in lectures, such as discussions around specific questions with random education peers, has been shown to increase course‐specific knowledge [31]. Within the laboratory setting, these transient interactions should be embedded within sessions regularly and could involve changing laboratory partners at the start of classes or during experimental incubations, and include tasks such as calculations, experimental design and data analysis. The information and skills developed in these mini‐breakout sessions could then be taken back to the original laboratory partner to be implemented or discussed further.

## Conflict of interest

The authors declare no conflict of interest.

## Author contributions

DPS and MML conceived and designed the project. SGC, HS and MML acquired the data. DPS and MML analysed and interpreted the data. DPS and MML wrote the paper.
